# NEEDS AND EXPERIENCES OF CAREGIVERS TO PERSONS WITH DEMENTIA WHO HAVE LOST THEIR DRIVING PRIVILEGES

**DOI:** 10.1093/geroni/igz038.414

**Published:** 2019-11-08

**Authors:** Stephanie Yamin, Cassandre Gratton

**Affiliations:** Saint-Paul University, Ottawa, Ontario, Canada

## Abstract

The ability to drive a motor vehicle for most older adults is associated with a sense of independence, well-being, quality of life and identity. For many older adults, driving cessation eventually becomes inevitable. This is especially the case for older adults with a diagnosis of dementia. Driving cessation has been shown to negatively impact individuals’ mobility and, consequently, quality of life. Informal caregivers (i.e., family caregivers) can mitigate the negative consequences associated with driving cessation in persons with dementia (PWD) by meeting their mobility needs and by offering emotional support. The purpose of this study was to examine the experience and needs of informal caregivers of PWD who had recently lost their driving privileges. Ten informal caregivers of PWD were recruited from a tertiary memory disorders clinic. Semi-structured interviews were conducted and transcribed. Transcripts of interviews were thematically analyzed using a grounded theory approach. The major themes emerging from the experience of caregivers included being overwhelmed by responsibility, overwhelmed by the emotional response of their care recipient and feeling resentment towards their care recipient. Similarly, the major themes emerging from the needs of caregivers included having the need for mobility training, psychoeducation on how to best attend to the emotional needs of their care recipient and the need for coping strategies. These experiences and needs expressed by caregivers indicate that driving cessation of the care recipient is a difficult experience for caregivers and that a therapeutic intervention based on the reported needs may be beneficial.

